# Therapy of Dredging the Bowels Enhanced the Neuroprotective Effect of Nourishing Kidney Herbs on Hippocampal Cholinergic System in Alzheimer's Disease Model Rat Induced by A*β* 1-42

**DOI:** 10.1155/2018/3282385

**Published:** 2018-09-12

**Authors:** Lu-Da Feng, Yang Tian, Xin Wang, Run Dai, Song Cai, Yu-Jia Cao, Yin-Chu Si

**Affiliations:** ^1^Dongzhimen Hospital, Beijing University of Chinese Medicine, Beijing 100700, China; ^2^Dongfang Hospital, Beijing University of Chinese Medicine, Beijing 100078, China; ^3^Neuroscience Department, Tufts University, Boston MA, 02111, USA; ^4^Research Center of TCM Information Engineering, Beijing University of Chinese Medicine, Beijing 100029, China; ^5^Department of Anatomy, School of Traditional Chinese Medicine, Beijing University of Chinese Medicine, Beijing 100029, China

## Abstract

**Background:**

Therapy of nourishing kidney has been used for treating memory deficits of Alzheimer's disease (AD) for thousands of years based on traditional Chinese medicine. However, we found the therapy of dredging the bowels could alleviate both memory deficits and mental symptoms of AD in clinic.

**Objective:**

To determine whether the therapy of dredging the bowels could enhance the neuroprotective effect of nourishing kidney herbs for treating AD rats, and to explore the underlying mechanism of the combination of nourishing kidney and dredging the bowels (NKDB) herbs.

**Methods:**

60 rats were randomly divided into sham-operated group (SOG), model group (MG), nourishing kidney group (NKG), dredging the bowels group (DBG), nourishing kidney and dredging the bowels group (NKDBG), and donepezil hydrochloride group (DHG). The model establishment was performed by injecting A*β* 1-42 into the hippocampal CA1 region. Animals received aqueous solution of Chinese herbal medicine or western medicine while SOG received only distilled water. Ability of learning and memory were assessed by Morris water maze. Acetylcholinesterase(AChE) and choline acetyltransferase (ChAT) activity and positive cells in the hippocampus were detected by the biochemical and immunofluorescent assay.

**Results:**

All rats were in the same baseline. While after model establishment, ability of learning and memory of MG, NKG, DBG, NKDBG, and DHG were significantly impaired compared with SOG. Whereas after treatment, ability of learning and memory of NKG, DBG, NKDBG, and DHG were significantly improved compared with MG. Additionally, AChE activity of NKG, DBG, and NKDBG was significantly decreased, meanwhile ChAT activity showed an increased tendency. The number of AChE-positive cells and ChAT-positive cells of both NKDBG and DHG were significantly decreased and increased respectively, superior to those when compared with NKG and DBG. What's more, there was no significant difference between NKDBG and DHG.

**Conclusion:**

Therapy of dredging the bowels could enhance the neuroprotective effect of nourishing kidney herbs by reversing morphological damage of hippocampal cholinergic system. Furthermore, treatment with NKDB herbs could be effectively against AD, providing a practical therapeutic strategy in clinic.

## 1. Introduction

Alzheimer's disease (AD) is a neurodegenerative disease and the most common cause of dementia [1–3]. It's estimated that 47 million people worldwide are living with dementia in 2016 and AD accounts for an estimated 60-80 percent, fatality and mortality rate [1, 4].

The clinical symptoms of AD are characterized by progressive memory deficits and cognitive decline, which may be due to neuronal and synaptic loss in the cerebral cortex and certain subcortical regions, especially hippocampus [5]. During the progression of AD, the death of neurons in the cerebral cortex leads to brain atrophy. In consequence, gaps develop in the temporal lobe and hippocampus where new information is stored and retrieved. These lesions affect the ability to remember, think, speak and make decisions [6]. In addition, acetylcholine (ACh) the neurotransmitter that plays an important role especially in memory is insufficient. ACh is synthesized and catalyzed by choline acetyltransferase (ChAT), then degraded by acetylcholinesterase (AChE). Related researches show that protein expression and activity of ChAT are reduced in the hippocampus of AD* in vivo* and* in vitro* [7–10]. Besides, AChE activity increases within and around amyloid-*β* (A*β*) plaques, leading to a decreased level of ACh in the cerebrum [11, 12].

Multiple hypotheses are currently advocated with regards to the primary cause of AD. However, A*β* cascade hypothesis that has been prevailing as a result of the senile plaque formed by extracellular A*β* aggregation is one of the major hallmark pathologies of AD [1]. A*β* fragment, especially A*β* 1-42 aggregation is considered as a critical protein which exerts neurotoxic effect, neuronal apoptosis and cholinergic dysfunction mentioned above [13]. Therefore, A*β* 1-42 administered rat is widely used as AD model for drug screening [14].

Although the patients' condition worsened gradually, current treatment options for AD are limited. U.S. Food and Drug Administration (FDA)-approved drugs are only symptomatic interventions, such as acetylcholinesterase inhibitors (AChEIs) and N-methyl-D-aspartate (NMDA) receptor antagonists, which are effective only for about half of the patients for approximately 6-12 months [15]. Under this serious circumstances, to find new therapeutic strategies is of vital importance.

Chinese herbal medicine (CHM) are commonly utilized for the prevention and treatment for central nervous system disorders based on traditional Chinese medicine (TCM), an important part of complementary and alternative medicine. Under the instructor of TCM theory recorded in the ancient book of* The Medical Classic of the Yellow Emperor (Huang Di Nei Jing)*, ‘the kidney nourishes marrow and brain is the sea of marrow', therapy of nourishing kidney has been used in treating AD for thousands of years with fewer adverse reactions [16, 17]. Related randomized, double-blind, placebo-controlled clinical trial of nourishing kidney herbs demonstrated that it could improve cognitive and physical function in AD patients [18]. Besides, it's reported that therapy of dredging the bowels with Dahuang (*Radix et Rhizoma Rhei*, RRR)-based CHM formula for treating mental disorders which are similar to mental symptoms of AD is effective [19–21].

Our previous researches demonstrate that nourishing kidney (NK) herbs could improve the disturbance in learning and memory by increasing the density of central cholinergic fibers, up-regulating the expression of brain-derived neurotrophic factor (BDNF) and tyrosine receptor kinase B (TrkB)-positive cells and their mRNA in the cortex and hippocampal CA1 region [22, 23]. Related animal experiments indicate NK herbs, the combination of Tusizi (*Semen Cuscutae*, SC) and Bajitian (*Radix Morindae Officinalis*, RMO) could increase superoxide dismutase activity and reduce malonaldehyde content in AD rat brain as well [24]. In addition, it's reported that dredging the bowels (DB) herbs including RRR, Zhishi (*Fructus Aurantii Immaturus*, FAI), Roucongrong (*Herba Cistanches*, HC), Maidong (*Radix Ophiopogonis*, RO), and Yujin (*Radix Curcumae*, RC) could significantly decrease AChE activity whereas increase ChAT activity and ACh content in the hippocampus of AD rats and act anti-aging effects [25, 26]. What's more, the combination of nourishing kidney and dredging the bowels (NKDB) herbs demonstrated potential against AD by up-regulating the expression of X-linked inhibitor of apoptosis protein [27]. However, it remains to be elucidated that whether NKDB herbs could affect hippocampal cholinergic system which is closely relevant to memory. Hence, this study is carried out to determine whether the therapy of dredging the bowels could enhance the neuroprotective effect of nourishing kidney herbs in treating AD and to explore underlying mechanism of the combination of NKDB herbs.

## 2. Materials and Methods

### 2.1. Ethics Statement and Experimental Schedule

**Figure 1:**
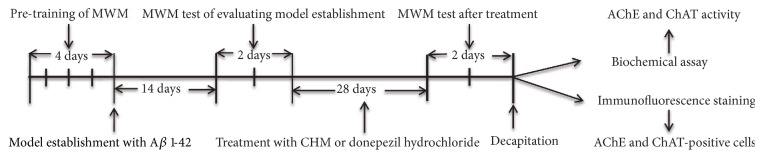
**Experimental schedule. **MWM: Morris water maze. CHM: Chinese herbal medicine.

Animal care and experimental procedures were performed according to the guide for the Care and Use of Laboratory Animals. The protocol was approved by the Joint Ethical Review Committee of Beijing University of Chinese Medicine (ID: BUCM-2-2016030301-1001). All efforts in surgeries were made to minimize suffering. The whole schedule of the model establishment, drug treatment, behavioral test, biochemical assay and immunofluorescence histochemistry assay are shown in Figure 1.

### 2.2. Animals and Grouping

A total of 60 clean grade healthy male Sprague Dawley rats (purchased from Beijing Weitong Lihua Test Animal Technology Co., LTD, Beijing, China), 2-month-old, 200 ± 20g, were housed in an air-conditioned room (21°C ± 2°C) under a 12-h light/dark cycle (lights on 07:00-17:00), with water and food available, in the Animal Laboratory of Science Experimental Center, Beijing University of Chinese Medicine. All rats were randomly divided into six groups by random number table, sham-operated group (SOG), model group (MG), nourishing kidney group (NKG), dredging the bowels group (DBG), nourishing kidney and dredging the bowels group (NKDBG), and donepezil hydrochloride group (DHG) with 10 rats in every group.

### 2.3. Model Establishment

A*β* 1-42 peptide (purchased from Sigma Aldrich, Saint Louis, USA) dissolved in dimethyl sulfoxide, was diluted with phosphate buffer saline (PBS) to the concentration of 2 *μ*g/*μ*l, and then was incubated at 37°C for 7 days for producing the aggregated form of peptide. The rats were fixed on a stereotaxic apparatus after narcotization with 10% chloral hydrate (3.5 ml/kg). Skin preparation was routinely performed on cranial, followed by disinfection was performed on the skin around surgery area. A 2-3 cm incision was cut along cranial midline and periosteum was exposed then. According to* < The Rat Brain in Stereotaxic Coordinates - 6th Edition >*, hippocampal CA1 region on the right side (AP -3.5mm, ML +2.0mm, DV -3.0mm from bregma) was set as injection target. Skull was drilled by dental auger, and then, a microscale injector was inserted into the drill hole for 5 min. 5 *μ*l solution was continuously injected into the target at a rate of 1 *μ*l/min for 5 min and needle was retained for another 5 min to guarantee the sufficient diffusion of the solution. The rats of MG, NKG, DBG, NKDBG, and DHG were injected with A*β* 1-42 at the concentration of 2 *μ*g/*μ*l while SOG were injected with the same amount of mixture of PBS and DMSO. The needle was then pulled out slowly, and injury around the hole drilled in the skull was disinfected with moderate penicillin powder. Finally, the skin was sutured.

### 2.4. Treatment with CHM or Western Medicine

CHM were divided into NK herbs, including SC and RMO, with the proportion of two herbs was 1: 1; DB herbs, including RRR, FAI, HC, RO and RC, with the proportion of five herbs was 0.6 : 1.2 : 1.5 : 1 :1; NKDB herbs, including all of the Chinese herbs with the same dosage mentioned above, and the proportion of seven herbs were 3: 3: 0.6: 1.2: 1.5: 1: 1. All CHM granules (purchased from Beijing Tcmages Pharmaceutical Co., LTD, Beijing, China) and Donepezil hydrochloride pills (purchased from Eisai pharmaceutical co., LTD, Suzhou, China) were dissolved in distilled water before gavage. The rats of NKG, DBG and NKDBG were given by gavage with NK herbs, DB herbs, NKDB herbs, at a dosage of 0.9 g/(kg·d), 0.88 g/(kg·d), 1.78 g/(kg·d) respectively while DHG were given by gavage with donepezil hydrochloride at a dosage of 0.5 mg/(kg·d), meanwhile, the rats of SOG and MG were given by gavage with the same dosage of distilled water. The medical treatment was set at the 15^th^ day after model establishment and was performed once a day for 4 weeks.

### 2.5. Morris Water Maze Test

The slight modification of Morris water maze (MWM) test was respectively performed before and after the model establishment and after medical treatment.

The experimental apparatus consisted of circular water tank (diameter 120 cm; height 45 cm) containing water and clouded with powdered black dye and maintained at 22 ± 2°C. The pool was geographically divided into four equally-sized quadrants (called quadrants I, II, III and IV) with a release point in every quadrant. A clear perspex platform (diameter 4.5 cm, height 14.5 cm) was positioned with its top submerged 2 cm below the water surface, in the center of quadrant III which was set as target quadrant. The trails of white rats in the black water pool were recorded as well as the data were analyzed by a video camera and an automated video tracking system device equipped with EthoVision XT 9.0 software.

*Pre-Training.* Before model establishment, all rats were arranged pre-training for 4 days. Every rat was remained on the platform for 20s to make sure where the platform located and then was released free into the pool facing towards the center of the pool at the release point of quadrant I, II and IV respectively. The maximum swimming time of the acquisition trial was 90s, and the rat would be guided to the platform and remained there for 20s following escape if it could not find the platform after 90s. The escape time spent by rat reaching the platform was recorded and termed as mean escape latency (MEL). Rats were dried and returned to the cages after training trial completed. 5 min gap was timed between the subsequent trials.

*Place Navigation. *Three detection point, after pre-training, 14^th^ day after the model establishment, and after the medical treatment respectively were arranged place navigation for 2 days. Every rat was released free into the pool, back towards the center of the pool, at the release point of quadrant I. MEL of all rats reaching the platform of 2 days were recorded and MEL was recorded as 90s if the rat couldn't find the platform after 90s.

*Spatial Probe.* All rats were arranged spatial probe on the first day after the place navigation. The platform in the pool was removed, and every rat was released free into the pool at the release point of quadrant I, back towards the center of the pool then. Frequency (F) of mice passing through the location where platform located before was recorded in 90s.

### 2.6. Preparation of Tissues Sample

After the final Morris water maze test, preparation of tissues sample came as followed. The rats were decapitated after narcotization. The hippocampus was taken out from brain tissue on the ice tray and weighted. Normal saline was added to the hippocampus tissue with the ratio of 1: 9 by weight (g): volume (ml), and the mixture was made into mechanical homogenate at a speed of 2500 r/min under the condition of ice water bath. Finally, the supernatant of the homogenate was extracted and stored at -80°C as the enzyme source to assay AChE and ChAT activity.

The brain tissue was obtained and processed according to standard protocols [28, 29]. Briefly, the rats were deeply anesthetized and then transcardially perfused with PBS about 200 mL until clear liquid drained out of the hole in the auricula dextra, followed by 300 mL of 4% paraformaldehyde in 0.1M PBS until the tail and limbs were twitched as well as the liver got harder. After the perfusion, the brains were then post-fixed in the same fixative overnight, followed by 30 % sucrose until sinking to the bottom, then stored at 4°C in preparation of immunofluorescence staining.

### 2.7. Biochemical Assay

Assay of AChE, ChAT activity of the hippocampus homogenates were performed by biochemical method with assay kit (purchased from Jiancheng Bioengineering, Nanjing, China). The absorbance was read by ultraviolet spectrophotometer. The protocol of biochemical assay in manufacture's instructions come as followed.

*AChE Activity. *Mix 0.5 ml substrate buffer and 0.5 ml of color buffer completely and incubate at 37°C for exactly 6 min. Add 0.03 ml inhibitor buffer and 0.1 ml hyalinize buffer into each tube. Put 50 *μ*l sample into contrast tube, then mix completely and stand the tubes for 15 min. After the inhibition, set the wavelength of the spectrophotometer at 412 nm and use 1 cm optical path cuvette. Use ddH_2_O to set zero. Test the absorbance of each tube and record. The activity of AChE in tissue (U/mgprot) = (testing  OD  –  contract  OD)/(standard  OD  –  blank  OD)× concentration of standard sample (1*μ*M) ÷ protein concerntration of testing sample (mgprot/ml).

*ChAT Activity*. After mixing reagents, prewarm in 37°C water bath for 5 min, then put in 25 *μ*l of the boiled supernatant of tissue homogenate and mix completely and incubate in the water bath for 20 min. After the incubation, 100°C boiled water bath to stop the reaction. Add in 425 *μ*l distilled water, mix completely. Centrifuge at 4000 r/min for 10 min. After the centrifuge, collect supernatant for color reaction. Mix 500 *μ*l supernatant with 10 *μ*l reagent G and stand the tubes completely and stand for 15 min. After the color reaction, set the wavelength of the spectrophotometer at 324 nm and use 1 cm optical path cuvette. Set zero with ddH_2_O and test the absorbance of each tube and record. The activity of ChAT in tissue (U/gprot) = (testing  OD  –  contract  OD)/(reacting  time  (20min) × 0.0198)  ×  total  volume  (600*μ*l)/sample  volume  (25*μ*l)  ÷  concentration  of  5%  tissue  homogenate  (gprot/ml)

### 2.8. Immunofluorescence Staining

The brains were dehydrated and embedded in paraffin. Continuous coronal sections were sliced by using microtome at a thickness of 5 *μ*m. Paraffin was removed by dimethyl benzene 3 times, 15 min per time, and then rehydrated with 100%, 95%, 90%, 80% and 70% graded ethanol to distilled water, with washes twice per step. Slices were rinsed in PBS (0.01 M, pH 7.2) 3 times, 3 min per time, followed by antigen retrieval under high pressure. The sections were blocked with 5% BSA, incubated for 20 min at 37°C. And then, the sections were respectively stained with rabbit-anti-rat primary antibody against AChE (purchased from Jiancheng Bioengineering, Nanjing, China) at a dilution of 1 : 70 with 5% BSA, and rabbit-anti-rat primary antibody against ChAT (purchased from Jiancheng Bioengineering, Nanjing, China) at a dilution of 1  :  70 with 5% BSA. After an overnight incubation at 4°C, the sections were rinsed with PBS 3 times, 3 min per time, and they were respectively stained with FITC-labeled goat-anti-rabbit IgG (purchased from Jiancheng Bioengineering, Nanjing, China) at a dilution of 1: 30 with 5% BSA. After incubation for 30 min, the sections were rinsed in PBS 3 times, 3 min per time, and stained with DAPI (purchased from Beijing Solarbio science and technology Co., LTD, Beijing, China). After being rinsed in PBS 3 times, 3 min per time, the sections were mounted with glycerin. Sections disposed with the same technique but without incubation with the primary antibody mentioned above were set as negative controls. All sections were observed under an Olympus microscope with confocal immunofluorescence.

### 2.9. Image Analysis and Statistics

The number of AChE and ChAT-positive cells of every group were automatically counted using ImageJ software in 4 random microscopic visions. All data were analyzed by using SPSS 20.0 statistical software and presented as the mean ± standard deviation, then converted as histogram using GraphPad Prism software 7.0. Data were analyzed by one-way ANOVA followed by LSD or Dunnett's comparison test. Differences were considered significant when* P* <0.05.

## 3. Results

### 3.1. Effects of NKDB Herbs on Learning and Memory Deficits Induced by A*β* 1-42

**Figure 2:**
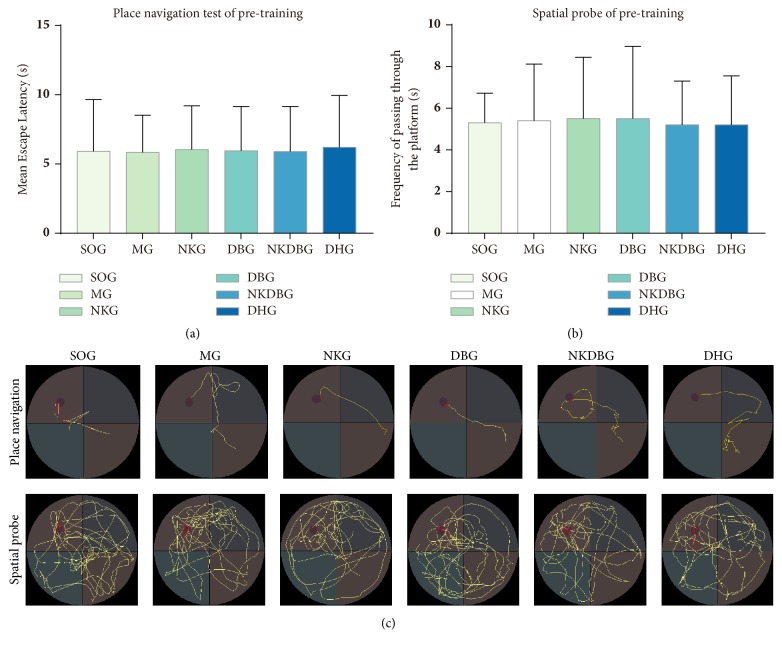
**Morris water maze test of pre-training before model establishment.** (a) Place navigation test in MWM. There was no statistical difference between every two groups. (b) Spatial probe in MWM. There was no statistical difference between every two groups. (c) Trails of rats respectively in place navigation test and spatial probe displayed search strategies were straight line type and valid.

After the pre-training, the test data displayed that there was no significant difference between all of the groups in MEL (F = 0.005,* P* > 0.05) and F (F = 0.028,* P* > 0.05) (Figures 2(a) and 2(b)). The trails displayed search strategy were straight line type and valid (Figure 2(c)). It suggested that experimental rats were in the same baseline before model establishment.

**Figure 3:**
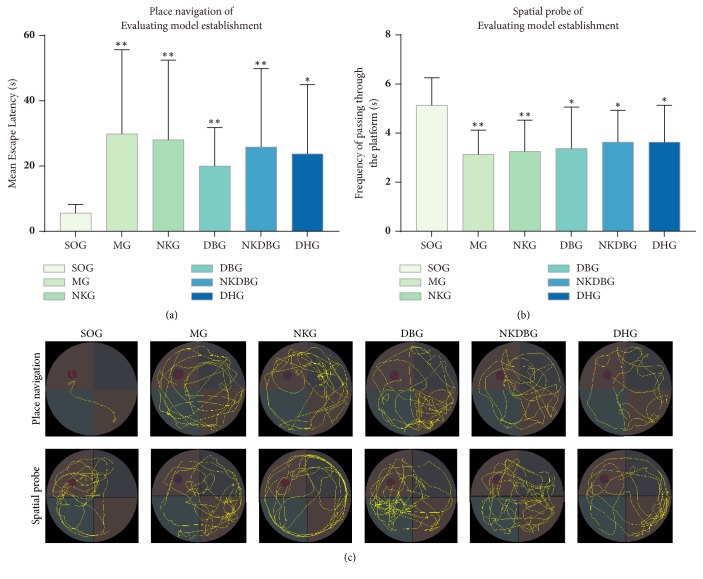
**Morris water maze test after model establishment induced by A** **β** **1-42. **(a) In place navigation test, compared with SOG, mean escape latency of MG, NKG, DBG, NKDBG, and DHG got significantly longer. (b) In spatial probe, compared with SOG, frequency of passing through the platform of MG, NKG, DBG, NKDBG, and DHG were significantly decreased. (c) Trails that transferred to random type and edge type displayed search strategies were invalid. Bar graphs were represented with the mean ± standard deviation (n = 10). ^*∗*^*P* < 0.05 and ^*∗∗*^*P <* 0.01 versus SOG.

After the operation, there were statistical difference in MEL (F = 1.208,* P* < 0.05) and F (F = 2.502,* P* < 0.05) between experiment groups. MEL of MG, NKG, DBG, NKDBG and DHG were respectively significantly longer than that of SOG (*P *< 0.05 or* P <* 0.01, Figure 3(a)); F were significantly decreased than that of SOG (*P *< 0.05 or* P <* 0.01, Figure 3(b)). The search strategy that transferred to random type and edge type were invalid (Figure 3(c)). It suggested that the model establishment was accomplished.

**Figure 4:**
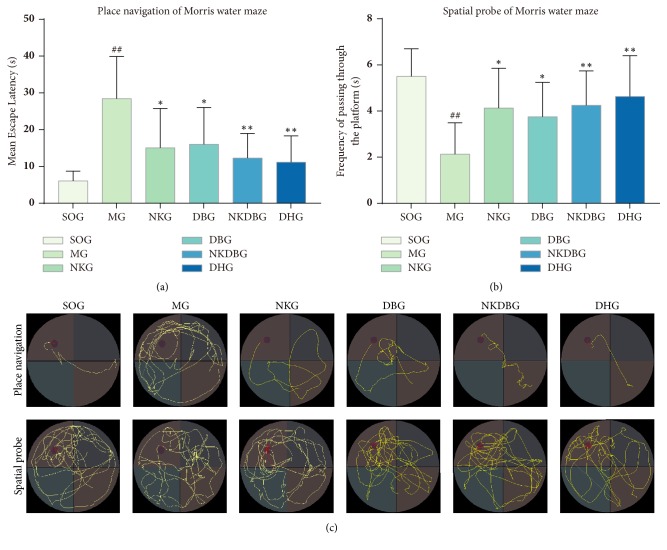
**Treatment with NKDB herbs ameliorated learning and memory deficits of AD rats induced by A** **β** **1-42.** (a) In place navigation test after treatment, mean escape latency (MEL) of MG were still significantly longer than that of SOG. While MEL of NKG, DBG, NKDBG, and DHG got significantly shorter compared with SOG. There was no statistical difference between NKDBG and DHG. (b) In spatial probe after treatment, frequency (F) of passing through the platform of MG were significantly decreased than that of SOG. While F of NKG, DBG, NKDBG, and DHG were significantly increased compared with MG. There was no statistical difference between NKDBG and DHG. (c) Trails that transferred gradually from edge and random type to tendency and straight line type displayed search strategies were optimized. Bar graphs were represented with the mean ± standard deviation (n = 10). ^##^*P* < 0.01 versus SOG, ^*∗∗*^*P <* 0.01 and ^*∗*^*P* < 0.05 versus MG.

After the treatment, there were statistical difference in MEL (F = 6.020,* P* < 0.001) and F (F = 4.193,* P* < 0.05) between experiment groups. MEL and F of MG were still significantly longer and decreased respectively compared with SOG (*P* < 0.01, Figures 4(a) and 4(b)). NKG, DBG, NKDBG and DHG showed significant improvement in MEL and F, respectively got shorter and increased (*P* < 0.05 or* P* < 0.01, Figures 4(a) and 4(b)) compared with MG. Besides, there was no significant difference between NKDBG and DHG. The search strategy were transferred gradually from edge and random type to tendency and straight line type (Figure 4(c)). It suggested treatment with NK herbs, DB herbs, NKDB herbs and donepezil hydrochloride for AD rats could improve learning and memory ability.

### 3.2. Effects of NKDB Herbs on AChE and ChAT Activity

**Figure 5:**
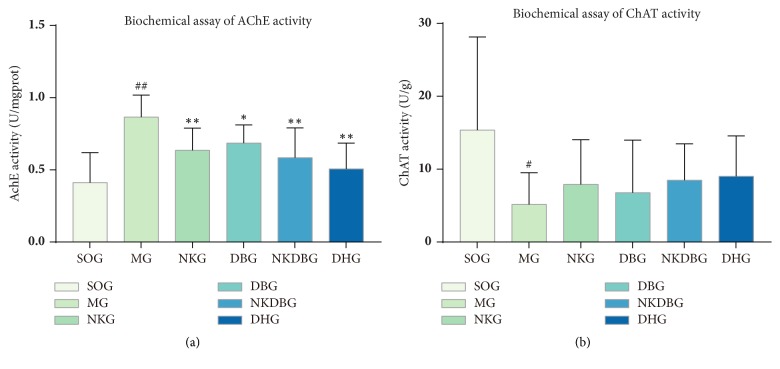
**Effects of NKDB herbs on AChE and ChAT activity in the hippocampus after the treatment.** (a) AChE activity of MG was significantly increased than that of SOG. Compared with MG, AChE activity of NKG, DBG, NKDBG, and DHG were decreased and the differences were statistically significant. There was no significant difference between NKDBG and DHG. (b) ChAT activity of MG was significantly decreased than that of SOG. There was a trend of increase in ChAT activity in NKG, DBG, NKDBG, and DHG compared with MG, without statistical difference. Bar graphs were represented with the mean ± standard deviation (n = 10). ^##^*P* < 0.01 and ^#^*P* < 0.05 versus SOG, ^*∗∗*^*P <* 0.01 and ^*∗*^*P* < 0.05 versus MG.

After the treatment, there were statistical difference in AChE activity (F = 6.399,* P* < 0.001) and ChAT activity (F = 3.091,* P* < 0.05) between experiment groups. On the one hand, there was a significant increase in AChE activity between MG and SOG. While AChE activity of NKG, DBG, NKDBG, and DHG was decreased when compared with MG, and the differences were statistically significant (*P *< 0.01, Figure 5(a)). On the other hand, ChAT activity of MG was decreased than that of SOG. However, there was a trend of increase in ChAT activity in NKG, DBG, NKDBG, and DHG compared with MG, without statistical difference (Figure 5(b)).

### 3.3. Effects of NKDB Herbs on the Number of AChE-Positive Cells and ChAT-Positive Cells

**Figure 6:**
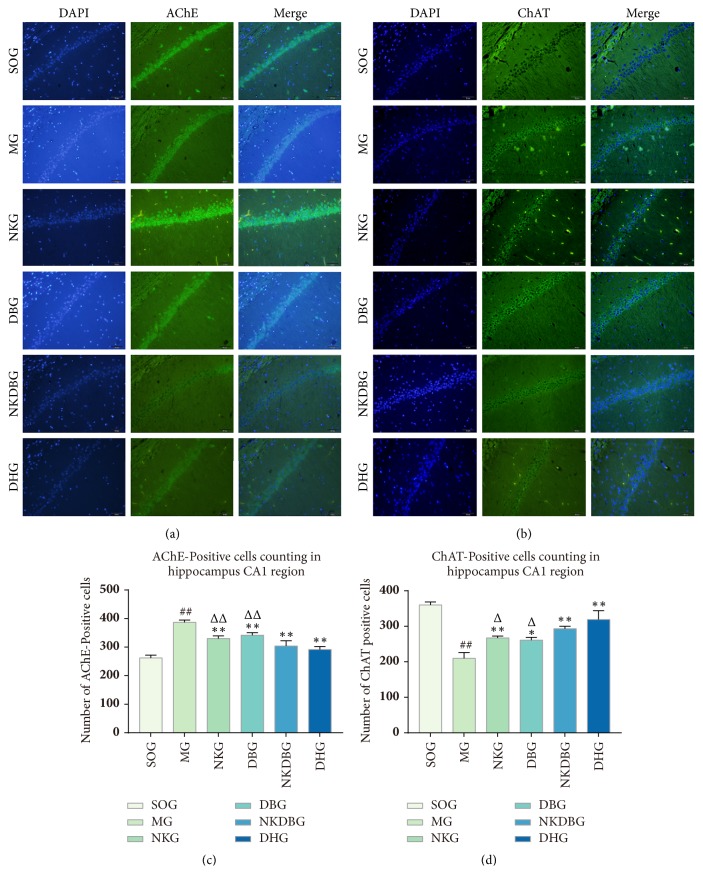
**Effects of NKDB herbs on the number of AChE-positive cells and ChAT-positive cells in hippocampal CA1 region after the treatment.** (a) Immunofluorescence staining of AChE-positive cells. (b) Immunofluorescence staining of ChAT-positive cells. (c) The number of AChE-positive cells. (d) The number of ChAT-positive cells. The sections were observed with magnification of 400× and scale bar was 50 *μ*m. Bar graphs were represented with the mean ± standard deviation (n = 4). ^##^*P* < 0.01 versus SOG, ^*∗∗*^*P* < 0.01 and ^*∗*^*P* < 0.05 versus MG, ^ΔΔ^*P* < 0.01 and ^Δ^*P* < 0.05 versus NKDBG.

The hippocampal structure could be clearly viewed. In CA1 region, AChE-positive cells were distributed in the synaptic cleft, and ChAT-positive cells were distributed in the synaptic terminal. In addition to neurons labeled were organized closely and regularly, nucleoli were hyperchromatic (Figures 6(a) and 6(b)). As analyzed, there were statistical difference in the number of AChE-positive cells (F = 58.004,* P* < 0.001) and ChAT-positive cells (F = 43.806,* P* < 0.001) between experiment groups. The number of AChE-positive cells and ChAT-positive cells in MG were respectively increased and decreased than those of SOG and the differences were significant (*P* < 0.01, Figures 6(c) and 6(d)), meanwhile, neurons labeled were scattered and there was obvious neuron loss. Whereas the number of AChE-positive cells and ChAT-positive cells in treatment groups were significantly decreased and increased respectively (*P* < 0.01 or* P* < 0.05, Figures 6(c) and 6(d)) compared with MG after the treatment. Furthermore, the changes of the number of AChE-positive cells and ChAT-positive cells in NKDBG were significant than those of NKG and DBG (*P* < 0.05, Figures 6(c) and 6(d)). And there was no significant difference between NKDBG and DHG. All data are not shown.

## 4. Discussion

### 4.1. Overview of the Study

The present study demonstrated that NKDB herbs ameliorated learning and memory deficits of A*β* 1-42 induced AD rats by means of increasing the number of ChAT-positive cells, decreasing AChE activity and the number of AChE-positive cells. MWM test was additionally arranged before the model establishment in order to eliminate differences caused by individual factors. What's more, both biochemical and immunofluorescence histochemistry assay were administered so as to observe the protective effects of NKDB herbs on cholinergic dysfunction in biochemical and morphology.

### 4.2. Neurotoxic A*β* 1-42 Leads to Memory Deficits

The cholinergic dysfunctions are accompanied by the occurrence of A*β* plaques deposed extracellularly in cerebral cortical and hippocampal regions. It has been hypothesized that A*β* peptides induce neurodegenerative changes at cholinergic terminals [30, 31]. The most predominant A*β* peptides detected in Alzheimer plaques are A*β* 1-40 and A*β* 1-42, whereas A*β* 1-42 displays higher neurotoxicity than A*β* 1-40. As major components of senile plaques in brains of AD patients, soluble A*β* oligomers are assumed to be the main neurotoxic species that induce neurotoxicity [32]. Thus, the model establishment in this study was administered by an aggregated form of A*β* peptide, A*β* oligomer, to induce neuronal damage and apoptosis, as other literature reported [33, 34].

In the present study, CA1 region of hippocampus was chosen as injection target with A*β* 1-42 and AD model was successfully established. MWM test showed that AD model rats did poor performance in place navigation and spatial probe respectively. It cost more time for AD model rats to find where platform initially located, and search strategy transferred to be invalid. It demonstrated that A*β* 1-42 leads to hippocampus damage and then cause the dysfunction of long-term memory and spatial memory, which is consistent with previous researches [14, 35].

### 4.3. NKDB Herbs Ameliorate Memory Deficits by Exerting Neuroprotective Effect against Dysfunction of Hippocampal Cholinergic System

Degeneration of cholinergic innervations in the septo-hippocampal pathway is believed as one of the common pathological features of AD [36, 37]. Central cholinergic neurons and critical neurotransmitter in cholinergic system, ACh, plays vital roles in learning and memory. As the key enzyme in cholinergic system, ChAT catalyzes the synthesis of ACh. Activity of ChAT directly reflects functional status of central cholinergic system. Besides, it is reported that impaired memory function is associated with a decrease of ChAT activity [14]. Another key enzyme in cholinergic system, AChE hydrolysis ACh and terminates its action in the synapse. Quantity and the activity of AChE are consistent with the quantity of ACh every nervous impulse needs. Current pharmacotherapy for AD is using AChEIs to increase ACh level through inhibition of AChE and improve AD symptoms by facilitating cholinergic neurotransmission [38]. According to the cholinergic hypothesis, memory deficits of dementia patients is due to irreversible deficiency of central cholinergic functions. ChAT and AChE are involved in the regulation of ACh to an adequate level. However, excessive AChE and lack of ChAT lead to ACh deficiency and cognitive decline [39]. In the present study, intrahippocampal injection with A*β* resulted in learning and memory deficits. And the memory deficits may be associated with decreased ChAT activity and number of ChAT-positive cells, increased AChE activity and number of AChE-positive cells in the hippocampus. Similar to previous reports, we infer that it is due to neuronal apoptosis and internal disturbance of hippocampal cholinergic system promoted by A*β* formation, subsequent aggregation and deposition according to A*β* cascade hypothesis [33, 34]. A*β* oligomer, assembly of A*β* fibrils, induces neuroinflammatory responses and ultimately leads to A*β* neurotoxicity in alteration of biochemistry and morphology of hippocampal cholinergic system. These pathological processes are in consistent with other reports [40–42]. Treatment with NKDB herbs which ameliorates memory impairment could increase the number of ChAT-positive cells, whereas decrease AChE activity and number of AChE-positive cells. Therefore, we confirm that the regulations of both enzyme activity and positive cells of ChAT and AChE are one of the underlying mechanisms of NKDB herbs, in accordance with previous studies about CHM formula or extract in treating AD [43–46].

### 4.4. Utilization and Characteristic of Chinese Herbal Medicine

CHM can be used by either single herb or polyherbal formula and the concept of poly-herbalism is peculiar to Oriental Medicine [47]. It is characteristic of CHM that each formula comprises several herbs with different or distinct functions that work synergistically at multiple targets of a complicated disease. The history of CHM indicates that they are generally safe unless administered in excessive doses, processed improperly or when they contain erroneous materials. NKDB herbs are composed of seven single herb mentioned above in appropriate dosage and they are widely used in CHM formulas based on TCM. Although NKDB herbs as compound formula haven't been qualitatively analyzed, main chemical constituents or content of effective components of every single herb are known.

Related in vivo or in vitro pharmacological studies of NK herbs and DB herbs come as followed. Extract of SC performed as neuroactive substance has been demonstrated to exert anti-aging effects by alleviating oxidative stress and to enhance the memory by inducing PC12 cell differentiation and decreasing AChE activity [48, 49]. RMO could prevent ischemic neurons damage by suppressing development of post-ischemic glucose intolerance and reverse oxidative damage caused by free radicals which are involved in AD [50–53]. Rhubarb aglycone extracted from RRR such as chrysophanol and emodin showed anti-inflammation with neuroprotective effect and effective inhibition on AChE activity [54, 55]. Aqueous FAI extracts significantly strengthened bowel movement by increasing the expression of 5-hydroxytryptamine receptor 4 and neurofilament-H in cathartic colons, the ultra-microstructure of which showed signs of neurodegeneration [56]. Glycosides extracted from HC enhances learning and memory deficits in AD model by depressing brain cells apoptosis rate, decreasing AChE activity, blocking amyloid deposition, reversing cholinergic and hippocampal dopaminergic neuronal function [57, 58]. RO could up-regulate superoxide dismutase gene expression [59]. Curcumin, phenolic compound extracted from RC could inhibit microglia cells inflammatory reaction caused by A*β* protein as well as reduce generation of A*β* protein [60].

## 5. Conclusion

In conclusion, the present study indicated that NKDB herbs effectively improve the ability of learning and memory in A*β* 1-42 induced rats. NKDB herbs are superior to NK herbs or DB herbs in the reversal of neuromorphological alteration. Thus, we infer that the therapy of dredging the bowels could synergistically enhance the neuroprotective effect of nourishing kidney herbs against A*β* neurotoxicity in neuromorphology. Present study sets the foundation for further research about application of NKDB herbs in the treatment of AD in future. Pathomechanism hypothesis of TCM theory, “Marrow damage due to kidney deficiency, Turbid lingering due to stagnation in the stomach and intestines” is deserved to be developed. Furthermore, we believe NKDB herbs used under the instructor of therapy of nourishing kidney and dredging the bowels could represent an important section and provide a reference for strategies in preventing and treating AD especially patients with obvious mental symptoms.

## Abbreviations

AD: Alzheimer's disease
A*β* 1-42: Amyloid *β* peptide 1-42
Ach: Acetylcholine
AChE: Acetylcholinesterase
ChAT: Choline acetyltransferase
AChEIs: Acetylcholinesterase inhibitors
TCM: Traditional Chinese medicine
CHM: Chinese herbal medicine
SOG: Sham-operated group
MG: Model group
NKG: Nourishing kidney group
DBG: Dredging the bowels group
NKDBG: Nourishing kidney and dredging the bowels group
DHG: Donepezil hydrochloride group
NK: Nourishing kidney
DB: Dredging the bowels
NKDB: Nourishing kidney and dredging the bowels
SC: * Semen Cuscutae*
RMO: * Radix Morindae Officinalis*
RRR: Radix et Rhizoma Rhei
FAI: * Fructus Aurantii Immaturus*
HC: * Herba Cistanches*
RO: * Radix Ophiopogonis*
RC: * Radix Curcumae*
BDNF: Brain-derived neurotrophic factor
trkB: Tyrosine receptor kinase B
ANOVA: Analysis of variance.
